# To cement or not to cement acetabular cups in total hip arthroplasty: a systematic review and re-evaluation

**DOI:** 10.1051/sicotj/2019032

**Published:** 2019-10-01

**Authors:** Frank Van Praet, Michiel Mulier

**Affiliations:** 1 Master of Medicine, KU Leuven Bergsken 50 9310 Moorsel Belgium; 2 Department of Development and Regeneration, KU Leuven Herestraat 49 3000 Leuven Belgium

**Keywords:** Total hip arthroplasty, Cemented versus hybrid, Revision rate, Functionality, Cost

## Abstract

*Introduction*: Total Hip Arthroplasty (THA) in the treatment of primary osteoarthritis of the hip has evolved to a very safe and cost-effective intervention with revision rates below 5% after 10 years. To this day, however, controversy remains on whether or not to cement the acetabular cup. *Methods*: A comprehensive PubMed search of the English literature for studies published between 2007 and 2018 was performed. Studies comparing the clinical (revision rate, functionality), radiological (wear) or economic (cost) differences between cemented (cemented stem with cemented cup) and hybrid (cemented stem with uncemented cup) prostheses for primary osteoarthritis of the hip were identified as eligible. *Results*: A total of 1032 studies were identified whereof twelve were included for qualitative synthesis. All studies concerning the risk of revision were based on registry data, covering a total of 365,693 cups. Cemented prostheses had a similar or lower risk of revision compared to hybrid prostheses in every study, but performed slightly worse on functionality and quality of life. While cemented prostheses were the cheapest option, hybrids were the most cost-effective. *Discussion*: The widespread preference for cementless fixation of the acetabulum cannot be explained by a superior survival of cementless or hybrid models. Irrespective of age, cemented fixation of the acetabulum remains the gold standard to which other techniques should be compared.

## Introduction

Total hip arthroplasty in the treatment of debilitating primary osteoarthritis of the hip has evolved to a very safe and cost-effective intervention with revision rates of less than 5% after 10 years [1, 2]. To this day, however, controversy remains on whether or not to cement the acetabular cup. Although the cementless cup, popular in western Europe and the United States, achieves better results each year, cementing the acetabulum remains, according to some, the gold standard to which any other Total Hip Prosthesis (THP) should be compared [1]. An extensive literature search could not identify a single systematic review comparing the results of cemented or cementless fixation of the acetabulum in the indication of primary osteoarthritis alone.

The introduction of the low-friction cemented polyethylene hip prosthesis by Professor John Charnley in 1962 marked a true revolution for hip surgery [3]. His low-friction model, based on an immediate cement fixation of a polyethylene cup, is currently approaching revision rates of 2% after 10 years [4], while the risk of revision was more than 1% per year in 1980 [5]. Despite changes in the chemical composition of cement, the techniques he introduced remain practically the same [2]. Out of fear for the “cement disease”, with the early occurrence of pelvic osteolysis [6], the popularity of cementless variants, for their fixation reliant on bone ingrowth on their rough surface, increased rapidly [7–9]. Based on the attractive idea of a biological integration into the bone and a shorter operating time of this technically less demanding fixation method, revision rates approximating those of a cemented cup are currently being achieved [4]. Although osteolysis was later proven to be the result of an inflammatory reaction to worn-out polyethylene debris (wear) [10], most Western countries almost exclusively use uncemented cups. The occurrence of osteolysis with subsequent aseptic loosening of the acetabular component remains the leading cause for late failure of a THP in Europe [11, 12].

This article is a systematic review on the risk of revision, wear and costs of cemented versus cementless cups in primary THA in the treatment of primary osteoarthritis of the hip. To analyse this, the so-called hybrid prosthesis (cemented stem with uncemented cup) and the fully cemented prosthesis (cemented stem with cemented cup) are ideal for assessing the isolated effect of cementing the cup. After systematic analysis of the literature, we will provide a critical re-evaluation of the value of cemented cups in THA, recognising that every surgeon relies on his own experience, knowledge and characteristics specific to each patient.

## Materials and methods

### Search strategy and identification of studies

**Figure 1:**
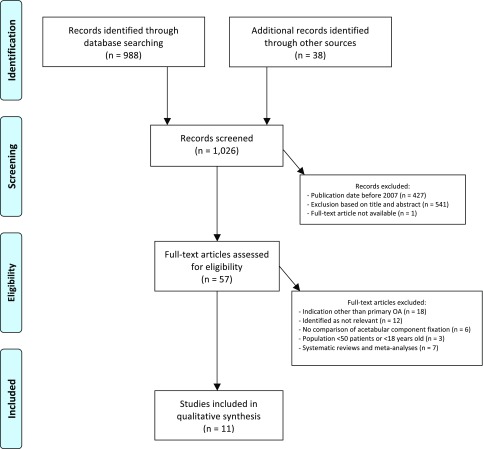
PRISMA Flow Diagram for selection and inclusion of relevant literature.

In accordance with the PRISMA checklist, an extensive search of the English literature was conducted in PubMed from 2007 to October 2018 (Figure 1). Studies comparing the clinical or radiological outcome of cemented and cementless cups were identified based on the following search terms, used in combination and separately: “total hip arthroplasty”, “cemented (vs.) uncemented”, “cementless”, “hybrid” and “revision rate”. An additional search for “cost” was conducted because of the special attention to this in this article. The reference list of each article was reviewed to identify additional relevant publications.

### Study selection

Based on title, abstract and ultimately entire article, the obtained literature was screened by a single reviewer. Various inclusion and exclusion criteria were established to minimise any potential selection bias. All studies comparing cemented with uncemented acetabulums for primary THA over a period of at least five years were considered eligible for inclusion. Outcome measures had to be of a clinical, radiological or financial nature. Exclusion criteria were THA for any indication other than primary osteoarthritis, patients less than 18 years of age and studies published before 2007. Given the differences in biology, treatment and prognosis in primary versus secondary osteoarthritis [13], studies with the latter indication were excluded. Both (systematic) reviews, meta-analyses, randomised controlled trials (RCT), prospective or retrospective cohort studies and registry data analyses underwent qualitative evaluation. Case reports and small patient groups (*n* < 50) were automatically excluded. In the rare case of multiple publication dates for the same article, the most recent article was chosen.

### Data extraction

The information extracted from each included study concerned study type, fixation methods, survival, number of procedures, patient characteristics, radiological and clinical outcome measures, complications, implants, place of performance and any conflicts of interest. Financial data were also obtained from both the articles and the Belgian National Database as available on the website of the “Technische Cel voor de verwerking van de gegevens met betrekking tot de ziekenhuizen”.

### Outcome measures

To estimate the real performance of each model as correctly as possible, statistical (survival percentages, revision rates), clinical (Patient Reported Outcomes Measures [PROMs], Harris Hip Score [HHS] [range 0–100], Oxford Hip Score [OHS] [range 0–48]; EQ-5D score for quality of life [range 0–1]) and radiological (migration, osteolysis; radiostereometric analysis and Livermore technique for wear) outcome measures were compared. In addition, since this review also highlights the financial aspect, the cost of the various models also formed an outcome measure.

## Results

The initial search yielded 988 results. An additional 38 relevant studies could be identified by reviewing the reference list of each article. After excluding 427 studies based on publication date and 541 studies based on title and abstract, one publication was excluded for interpretation due to unavailability of the full text. Of the 57 remaining studies, 18 were subsequently excluded due to a different indication than primary osteoarthritis. Twelve studies were assessed as irrelevant, six studies did not make a comparative analysis of the acetabular component and three studies did not meet the population criteria for inclusion. Finally, seven systematic reviews and meta-analyses were excluded because they were based on studies published before 2007.

A total of eleven studies remained for qualitative synthesis. Of these, seven discussed survival and risk of revision after cemented and cementless fixation of the acetabulum. Four also included an economic analysis in their work. Only one study on the occurrence of wear in cemented versus cementless cups met all the conditions for inclusion. All seven studies discussing survival were based on registry data (in total 365,693 cups, at least five years of follow-up) and looked at the risk of revision after fully cemented and hybrid THA for primary osteoarthritis. Of these, two also analysed survival after cementing (fully cemented THP) or not cementing (hybrid and cementless THP) of the acetabulum alone. A distinction was made between revision for any reason and revision for aseptic loosening. When only survival rates were being displayed, revision rates could easily be calculated.

### Revision for any reason

When comparing cemented with hybrid models, the risk of revision for any reason was not significantly different in five [4, 14–17] and significantly smaller for cemented models in two [18, 19] studies. In these last two studies, results were significantly more favourable for cemented than for hybrid prostheses, irrespective of age. In patients aged 35–55 years, there were 65% more revisions (HR 1.7; 95% CI [1.2–2.3]) during the first two years for hybrid compared to cemented implants, after which this effect decreased to 35% (HR 1.3; 95% CI [1.2–1.5]). This effect remained significant for up to 16 years postoperatively. In this study, dislocation was the main reason for revision during the first two years [18]. A second study found the same significant effect five years after the procedure (HR 1.26; 95% CI [1.12–1.42]; *p* < 0.001) [19]. For the five remaining studies, the trend also was slightly more favourable for cemented prostheses, but no significance levels were achieved. Not a single study could show a significantly lower risk of revision for any reason for hybrid compared to cemented models.

When comparing all the cemented with all the cementless cups (implanted in cemented and both hybrid and cementless models, respectively), a single study concluded that there was a significantly higher risk of revision during the first year after cementless fixation of the cup in patients more than 80 years of age (HR 1.41; 95% CI [1.10–1.81]; *p* = 0.007). However, this “protective” effect of a cemented socket was no longer present five years after the procedure (HR 0.41; 95% CI [0.10–1.69]) [17].

### Revision for aseptic loosening

Only two studies reported revision rates for aseptic loosening of the prosthesis. A first study could not show any difference between cemented and hybrid models in the annual risk of revision for aseptic loosening up to 16 years postoperatively (HR 1.91; 95% CI [0.81–4.50]) for the youngest patient group studied (35–55 years) [18]. When comparing all the cemented with all the cementless cups, however, a second study proved that cementless cups have significantly lower revision rates for aseptic loosening in patients over 55 years of age (RR 0.54; 95% CI [0.47–0.62]; *p* < 0.001) [15]. A complete overview of the revision rates for any reason and for aseptic loosening is shown in Table 1.

**Table 1:** Comparison of revision rates for cemented and hybrid total hip arthroplasty for primary osteoarthritis, categorised by age group.

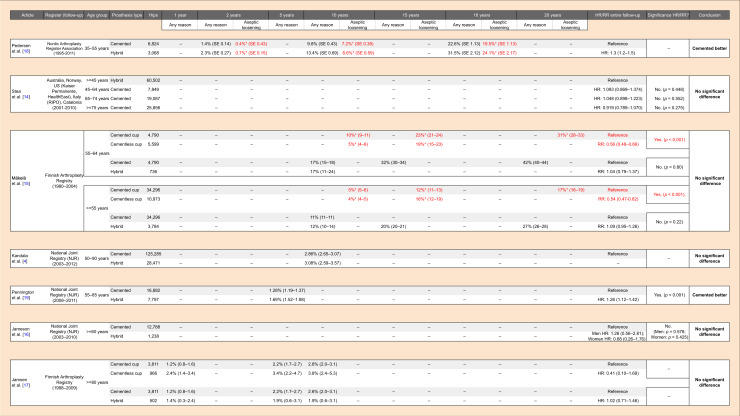

Grey-coloured cells were used as a reference in risk calculations. 95% confidence intervals are shown between brackets (– = not reported; * = revision for aseptic loosening, coloured red. CI, confidence interval; SE, standard error; HR, hazard ratio; RR, relative risk).

### Wear

Only a single study with 92 participants made a direct comparison between the occurrence of wear in cemented and cementless acetabulums [20]. The total amount of wear after 10 years was on average 1.07 ± 0.78 mm in cemented cups, compared to 1.18 ± 0.61 mm on average in cementless cups. Although these values did not differ significantly from each other (*p* = 0.437), they did find significantly more wear in patients with a younger age (*p* = 0.003) and the male gender (*p* = 0.003). It is important to note that they chose to place cemented cups in all patients more than 70 years of age, while every patient less than 60 years of age received a cementless cup.

### Functionality

A total of three studies analysed differences in functionality after cemented or hybrid THA based on data from the English National Health Service (NHS). In all studies, cohorts were matched or corrected for preoperative differences in age, gender and functionality, among other things. Two of these [19, 21] concluded that hybrids resulted in a better functionality and quality of life. A third study [16] was unable to demonstrate a significant difference in functionality. When comparing the most commonly used brands of prostheses within both cemented, hybrid and cementless models [19], all hybrid prostheses were identified as significantly best performing in terms of functionality and quality of life (*p* < 0.001), despite the small but significantly lower revision rates of cemented prostheses (*p* < 0.001). In this first study, after correction for preoperative differences, the average functionality, measured six months postoperatively, was best for hybrid (OHS 39.4; 95% CI [39.1–39.7]) and worst for cemented models (OHS 37.7; 95% CI [37.3–38.1]). There was also a higher quality of life with hybrid (EQ-5D 0.80; 95% CI [0.79–0.81]) than with cemented (EQ-5D 0.76; 95% CI [0.75–0.77]) prostheses. After subgroup analysis, within the hybrid brands, there appeared to be no significant difference in postoperative functionality, while there was a significant difference in terms of survival, with the Exeter V40 Trident having the lowest risk of revision after five years (1.34%; 95% CI [1.06–1.69]). As no adjustments were made for preoperative differences when comparing this specific brand with other brands, this percentage was not included in the comparison of the revision rates in Table 1. A second study [21], based on the same English database, concluded that for women of 70 years of age, the quality of life was higher after hybrid compared to cemented fixation, with higher average EQ-5D scores (0.81 vs. 0.78) and higher Quality Adjusted Life Years (QALYs) (9.3 vs. 9.0) after hybrid fixation. This difference was no longer present among women aged 80, where cemented prostheses scored slightly better (EQ-5D 0.751 vs. 0.754). A final study [16] concluded that in patients more than 60 years of age, postoperative functionality was independent of the fixation method used. Both for men and women, no significant difference could be demonstrated in the improvement of postoperative functionality as measured by the OHS score (*p* values 0.140 and 0.207, respectively) and EQ-5D score (*p* values 0.302 and 0.312, respectively).

### Economical evaluation

Three NJR-based retrospective cohort studies [16, 21, 22] and one review [23] discussed the economic aspects in the choice of whether or not to use cement. Of these, two [16, 21] considered the differences in cost after cemented or hybrid and two [22, 23] after cemented or completely cementless THA. Taking into account material costs and costs associated with differences in revision rates, the cemented prostheses were the cheapest option in every single article. Considering the better quality of life and QALYs already described after hybrid fixation, a cost-effectiveness analysis could calculate that the additional cost per QALY in patients aged 55–84 compared to cemented THA was 2500 pounds [21]. Eighty-year-old ladies were an exception to this, with cemented prostheses being the most cost-effective. The cost for the most commonly used cemented THP (Exeter V40 stem with cemented Contemporary cup) was 1138.09 pounds, while it was 2160.11 pounds for the most commonly used hybrid THP (Exeter V40 stem with cementless Trident cup) [16]. Although the cement itself was an additional cost for cemented THA (107 pounds for four cement mixes), this cost was relatively small compared to the much more expensive components (polyethylene liner and metal shell) used for cementless fixation of the acetabulum [16]. When comparing cemented with fully cementless THA, a third study [22] showed that if in England all hip prostheses would be cemented, 10 million pounds in material costs would be saved each year, with an additional saving of 8.5 million pounds in the five following years because of the lower risk of revision after cemented compared to cementless THA. In this study, the average cost of a cemented acetabular component was 285 pounds (79–1077 pounds) and that of a cementless component was 511 pounds (100–1288 pounds). The additional cost for cementing the acetabulum (the cement itself and the required materials) was calculated to be 55 pounds. A final review article [23] observed that with each operation, 15–20 min could be saved if cementless components were used instead of cemented components, resulting in a saving of 150–200 pounds per procedure.

## Discussion

Despite the excellent results of both cemented and cementless prostheses, controversy remains as to whether or not to cement the acetabular cup in primary THA. This systematic review takes into account clinical, radiological and economic factors in the choice of whether or not to cement the acetabulum. Previous reviews were not limited to primary osteoarthritis of the hip alone.

### Revision rates

Regardless of the patient’s age, cemented prostheses have a lower risk of revision for any reason. Although significance levels were not achieved in every study, the trend was practically the same in every study. The overall lower risk of revision after cementing the cup, both in the short and the long term, underscores the need to reopen the discussion about the use of cement. In line with previous research, even after the first postoperative decade, a more reliable fixation is achieved when cementing the acetabulum [24]. The described advantages of cementless cups such as a longer lasting fixation due to bone ingrowth, shorter operating time [23], better functionality and the relative technical simplicity [25] are not reflected in a lower risk of revision. A more difficult to quantify but all the more relevant aspect in the interpretation of these results is that personal preference, experience, patient characteristics and prosthesis availability are important factors that will play a role in the choice of and results with a certain type of fixation [26].

### Wear

The described higher wear rates after cementless fixation of the acetabulum [27, 28] are also suggested in this study, but are based on two unmatched cohorts and do not reach a level of significance. It is therefore difficult to draw hard conclusions based on these data. Osteolysis due to polyethylene wear and subsequent aseptic loosening of the prosthesis remains the dominant limiting factor in achieving long-term fixation for both models [29]. Putting maximal effort into minimising the amount of wear can therefore be justified perfectly. Biomechanical differences in load transfer to the periacetabular bone and the occurrence of backside wear due to micromotion of the polyethylene liner in the metal acetabular shell of cementless modular cups are partly the cause for the difference in wear between the two methods of fixation [30]. Despite the slightly lower wear rates of cemented cups, they are more often revised due to aseptic loosening, which seems to contradict the causal relationship between wear and aseptic loosening described above [31]. However, wear is not the only cause for osteolysis [32]. In addition to micromovements between bone and prosthesis, the (highly) cross linking of polyethylene is also a determining factor for the degree of osteolysis due to a difference in osteolytic potential [32, 33]. Despite the conflicting conclusions with revision rates for any reason, survival tables based on aseptic loosening are often misleading and of limited value [28]. However, this does not alter the fact that a further commitment to minimising the amount of wear is required. Monoblock elastic cups (cementless polyethylene cups without a metal shell) and vitamin E-incorporated cups or combinations of both (RM Pressfit Vitamys cup) are already delivering promising results [34, 35].

### Functionality

Cementless cups as represented in hybrid models give patients a slightly better postoperative functionality and quality of life. This is in line with previous research in England, which, based on 24,709 patients, was able to demonstrate a better functionality for cementless and hybrid components in patients under the age of 60 [36]. It should be noted that patients who underwent cementless fixation of the acetabulum had, on average, slightly higher preoperative functional scores. Due to the ceiling effect of both OHS and EQ-5D scores, the demonstration of a real functional superiority can be difficult.

### Cost-effectiveness

In the future, not only differences in revision and wear rates, but also differences in cost-effectiveness will guide further developments [2]. Although cemented parts are clearly cheaper than hybrid parts, this extra cost is justified by some proponents by a shorter operating time [23]. However, this argument is only valid if additional procedures can be scheduled within an operating schedule [16]. The savings associated with a slightly shorter operating time also pale into insignificance compared to the savings that can be made by a less frequent need for revision and shorter postoperative stay [23]. For example, in 2016 in Belgium, the average total cost of placing a hip prosthesis was 9668.56 euros per hospitalisation [37]. The superior functionality and associated higher cost-effectiveness after implantation of a hybrid prosthesis, on the other hand, have an important weight within an increasingly personalised medicine. After all, the priorities of an active 50-years-old man often lie elsewhere than those of an 80-years-old woman.

### Critical note

Not all bearing-type combinations provide equal results after being cemented. As it is not common practice to cement a ceramic-on-ceramic implant, they are excluded by some authors. Furthermore, the need to achieve a high degree of technical perfection when placing a cemented cup is cited by some as a reason to declare this technique as obsolete [25]. It is important to note, however, that long-term results are significantly influenced by a surgeon’s experience with a certain type of fixation [12]. The decreasing popularity of cemented components is in that respect detrimental to adequately teaching this technically more challenging technique to the youngest generation of surgeons. In addition, the widespread assumption that a “simple” polyethylene exchange of a well-fixed cementless modular cup is a benign procedure [28] can lower the threshold for performing a revision, after which the risk of a second revision is significantly increased [38]. However, this increased risk of re-revision does not alter the fact that even for 80-year-olds major improvements in functionality are achieved after revision of a cementless cup [39]. Accordingly, with the increasingly effectiveness-driven financing of healthcare in mind, the reported better functionality and cost-effectiveness of hybrid models make a good argument for their use. Finally, the use of cement at the acetabular site does not seem to cause a rise in postoperative mortality, while cementing the femoral stem increases this risk only slightly [40].

**Figure 2:**
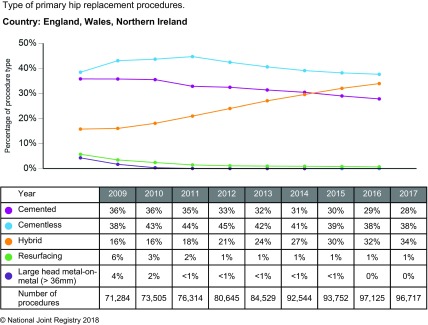
Types of prostheses used for primary THA in England, Wales and Northern Ireland. National Joint Registry (NJR). Types of primary hip replacements undertaken [Internet]. 2018. Available on: http://www.njrreports.org.uk/.

Although this study did not focus on fully cementless models (cementless stem with cementless cup), they were analysed in almost every included study. Interestingly, not a single study decided that cementless models were superior compared to cemented or hybrid models, both in terms of risk of revision and cost-effectiveness. The increasing use of cementless components, both in England and in the Scandinavian countries ([41], Figure 2), is attributed by some authors to the marketing strategies of implant manufacturers [42].

### Strengths and weaknesses

A strength of this systematic review is that confounders associated with whether or not cementing the stem could be eliminated by comparing cemented with hybrid models. Nonetheless, this review is subject to the strengths and weaknesses inherently connected to each database study. Differences in revision rates between different models are often small. The high statistical power that can be achieved with registry data is an important advantage in that respect [43]. Moreover, databases reflect the results as they occur in an entire community and are less subject to any potential conflict of interest [44].

The main shortcoming of registries is the fact that they only contain observational data and are thus subject to multiple forms of bias. For example, a poorly performing implant might only be used by a limited number of surgeons, while it might perform better in other hands [43]. By applying strict inclusion and exclusion criteria, only a limited number of studies remained for qualitative synthesis. The fact that only registry data analyses could be included for the risk of revision underscores the need for good randomised studies with specific focus on primary osteoarthritis alone.

## Conclusions

Both cemented and hybrid models provide excellent results in both the short and the long term. However, the widespread preference for cementless fixation of the acetabulum cannot be explained by a superior survival of cementless or hybrid models. Irrespective of age, cemented fixation of the acetabulum remains the gold standard to which other techniques should be compared.
